# Computational and Experimental Approaches for Determining Scattering Parameters of OPEFB/PLA Composites to Calculate the Absorption and Attenuation Values at Microwave Frequencies

**DOI:** 10.3390/polym12091919

**Published:** 2020-08-26

**Authors:** Ahmad Fahad Ahmad, Sidek Hj Ab Aziz, Zulkifly Abbas, Daw Mohammad Abdalhadi, Ahmad Mamoun Khamis, Umar Sa’ad Aliyu

**Affiliations:** 1 Department of Physics, Faculty of Science, Universiti Putra Malaysia, UPM Serdang 43400, Selangor, Malaysia; sidek@upm.edu.my (S.H.A.A.); akhameis@yahoo.com (A.M.K.); 2 Department of Physics, Faculty of Science, Al Asmarya Islamic University (AIU), Zliten 218521, Libya; doibnsahl@yahoo.com; 3 Department of Physics, Faculty of Science, Federal University Lafia, Nasarawa Sate 950101, Nigeria; usaltilde@yahoo.com

**Keywords:** attenuation, absorption, reflection, transmission, microstrip, COMSOL

## Abstract

This article describes attenuation and absorption measurements using the microstrip transmission line technique connected with a microwave vector network analyzer (Agilent 8750B). The magnitudes of the reflection (S_11_) and transmission (S_21_) coefficients obtained from the microstrip transmission line were used to determine the attenuation and absorption of oil palm empty fruit bunch/polylactic acid (OPEFB/PLA) composites in a frequency range between 0.20 GHz and 12 GHz at room temperature. The main structure of semi-flexible substrates (OPEFF/PLA) was fabricated using different fiber loading content extracted from oil palm empty fruit bunch (OPEFB) trees hosted in polylactic acid (PLA) using the Brabender blending machine, which ensured mixture homogeneity. The commercial software package, Computer Simulation Technology Microwave Studio (CSTMWS), was used to investigate the microstrip line technique performance by simulating and determine the S_11_ and S_21_ for microwave substrate materials. Results showed that the materials’ transmission, reflection, attenuation, and absorption properties could be controlled by changing the percentage of OPEFB filler in the composites. The highest absorption loss was calculated for the highest percentage of filler (70%) OPEFB at 12 GHz to be 0.763 dB, while the lowest absorption loss was calculated for the lowest percentage of filler 30% OPEFB at 12 GHz to be 0.407 dB. Finally, the simulated and measured results were in excellent agreement, but the environmental conditions slightly altered the results. From the results it is observed that the value of the dielectric constant ($\varepsilon_{r}^{\prime })$ and loss factor ($\varepsilon_{r}^{\prime \prime })$ is higher for the OPEFB/PLA composites with a higher content of OPEFB filler. The dielectric constant increased from 2.746 dB to 3.486 dB, while the loss factor increased from 0.090 dB to 0.5941 dB at the highest percentage of 70% OPEFB filler. The dielectric properties obtained from the open-ended coaxial probe were required as input to FEM to calculate the S_11_ and S_21_ of the samples.

## 1. Introduction

In recent years, the research and development of environment-friendly polymers have attracted more attention due to the concerns related to environmental pollution by non-degradable plastic wastes. Natural fiber-reinforced composites using thermoplastic such as polypropylene and polyethylene as a matrix have been widely used in automotive applications, but the composites are partially biodegradable. Thus, the formulation of composites with matrices, which originate from biodegradable raw material such as polylactic acid, should be investigated [1]. Since polylactic acid (PLA) is environment-friendly, it became one of the polymers in the highest demand to be used in applications that are difficult to be recycled. PLA is commonly used to replace commodity synthetic polymers that can cause deterioration of our environment due to solid waste pollution. Many studies on the mechanical properties of natural fiber reinforced with PLA have been developed by other researchers with different types of fiber, including kenaf [2], jute [3], bamboo, [4], ramie [5], banana [6], oil palm [7], and other natural fibers studied as reinforcements to replace synthetic fiber in polymer composites. Nishino et al. [8] performed a study on the retted long fiber as reinforcement using PLA polymer as a matrix. They found that fiber loading, up to 70%, provided impressive mechanical properties comparable to traditional composites. Other studies focused more on molding conditions, mechanical properties of the kenaf fiber biocomposite materials, and interfacial bonding of the biocomposites prepared. Thus, reinforcing with natural fibers is one possibility of reducing the cost, improving stiffness, and enhancing thermal stability.

Oil palm empty fruit bunch (OPEFB) fiber is one of the major solid wastes produced by the oil palm industry. However, the primary drawback of using natural fibers for reinforcement is the poor interfacial adhesion between polar-hydrophilic OPEFB and non-polar-plastics. Oil palm fiber is of low-cost, relatively high strength and stiffness, low density, and causes no skin irritations after coming in contact with it as it can be used as alternative to synthetic fibers in environmental applications [9,10]. Polymer composites are a major component in the design of new advanced materials appropriate for a variety of applications, such as high-frequency devices and electrical engineering devices. The reinforcement of polymers using various types of organic or inorganic fillers is a popular practice in the production of systems to improve thermal conductivity and meet mechanical requirements and dielectric properties [11]. Characteristics of the polymer composites are influenced by many factors, such as intrinsic characteristics of each component, shape, kind of filler, the dimension of the fillers, and the nature of their interfaces, percentages of filler, frequency, permittivity, and permeability [12]. The behavior of considered materials is very important when placed in an electromagnetic field, especially when it relates to military hardware, electronics, communication, and industrial applications and shielding [13,14].

There has been very limited research reported on electrical applications of natural fiber-reinforced polymer composites and their electrical properties. The electrical characteristics of PLA were reported by [15], showing that in the range from room temperature to about 70 °C, PLA offers excellent insulation properties, such as high resistivity and high breakdown strength, comparable to the low-density of petroleum-derived plastics. Daw et al., 2019 [16], have studied the effectiveness of the various ratios of Fe_2_O_3_ onto dielectric and magnetic properties of a Fe_2_O_3_ OPEFB/PLA composite at a frequency range of 8–12 GHz. The results of the electromagnetic properties indicate that the permittivity and permeability increased by increasing the Fe_2_O_3_ percentage. Furthermore, the power loss and absorption loss increased with both the increase in frequency and the percentage of Fe_2_O_3_ filler in the composite. The findings also showed that the material transmission, reflection, and absorption properties can be controlled by changing the percentage of Fe_2_O_3_ filler in the composites. Most studies on the microstrip line involve measurements of the transmission coefficient of materials in the microwave frequency range of 4 GHz with samples openly placed on the strapline [13].

Khamis et al., 2020 [17], have studied the dielectric properties, transmission coefficient S_21_, reflection coefficient S_11_, reflection loss, and power loss for the epoxy resin (ER) reinforced with different percentages of micro-sized oil palm empty fruit bunch (OPEFB) at a frequency of 8–12 GHz. The reflection and transmission coefficients of the composites were measured using a rectangular waveguide connected to the vector network analyzer. The results showed that the dielectric properties increased, while S_11_ and S_21_ decreased with the OPEFB percentage increasing in the composites. Furthermore, the shielding effectiveness, power loss, and reflection loss increased with increasing OPEFB percentage. The simulated and measured results of S_11_ and S_21_ were in good agreement. The scope of this study includes the utilization of the microstrip transmission line technique in the measurement of reflection S_11_ and transmission S_21_ coefficients in the frequency range between 0.20 GHz and 12 GHz to determine attenuation and absorption values for OPEFB/PLA composites, as well as a systematic comparison between the experimental results and the theoretical (simulation) results obtained through the application of the COMSOL software.

## 2. Experimental Details

### 2.1. Materials

**Figure 1:**
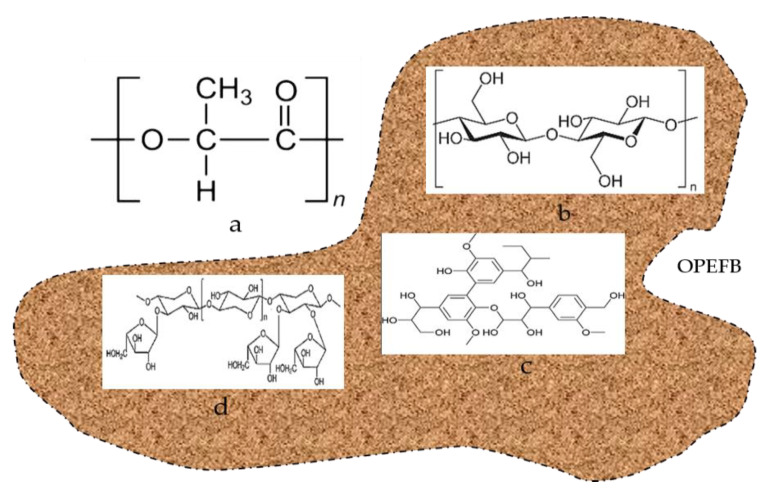
Chemical structure of (**a**) polylactic acid (PLA) and chemical structure of oil palm empty fruit bunch (OPEFB) fiber content include (**b**) cellulose, (**c**) hemicelluloses, and (**d**) lignin.

The materials used in this research include OPEFB fiber obtained from, (Sabutek (M) Sdn, Selangor, Malaysia), Poly(lactic acid) pellets with density 1.24 g/cm^3^ (Grade 4060D) was supplied by Nature Work LLC (Minnetonka, MN, USA), and the COMSOL Multiphysics 3.5 software (COMSOL Multyphysics, Burlington, MA, USA) was utilized in the theoretical simulation of the microstrip results by calculating the Scattering (S)-parameters coefficients of the samples. The RT-Duroid 5880 substrate (manufactured in the USA) was used to place the microstrip layout, and the ferric chloride (FeCl_3_) solution from Nature Work LLC (Minnetonka, MN, USA) was used for etching purposes (immersing the microstrip board). Figure 1 depicts the chemical structures of PLA and OPEFB.

### 2.2. Microstrip Transmission Line Fabrication

**Figure 2:**
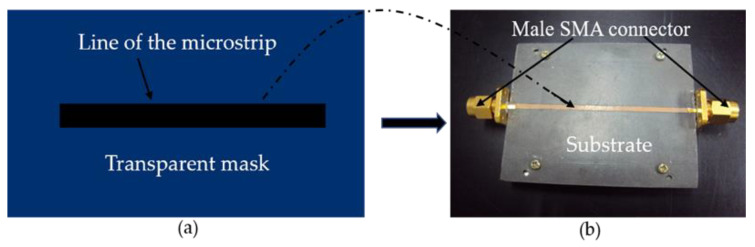
(**a**) Microstrip transmission line printed on a transparent mask. (**b**) The complete microstrip straight line.

The proceedings to prepare a microstrip transmission line utilized in this study are depicted in Figure 2a,b. The microstrip fabricated using the RT-Duriod 5880 as a substrate has complex permittivity (2.2-j*0.00088), aluminum, connectors, and a transparent plastic sheet. The transparent mask was carried out by the Auto CAD2001 software)Portland, OR, USA( in conjunction with a high-resolution laser printer (HP LaserJet 1200, Hewlett-Packard, CA, USA) to help transfer the initial figure to the real figure of the sensor [18]. The substrate was thoroughly cleaned from dust, grease, and particles, or the contaminants were removed by rinsing and drying. The substrate had a dimension of 60 mm × 50 mm and a thickness of 0.7874 mm, while the transmission line had a length of 60 mm, width of 1.5 mm, and a characteristic impedance of >50 Ω. The transmission line, as shown in Figure 2a, was placed on the surface of the substrate, covering it with a piece of cloth, then pressed using a hot iron all over the substrate. Etching was achieved by immersing the board in the solution prepared by adding 1 kg of ferric chloride (FeCl_3_) to a liter of water. The etching process time can be shortened by heating the solution to 50 °C. The purpose of etching is to remove the unwanted part of the metal (copper), leaving only the designed circuit. Next, the substrate was placed on an aluminum slab, giving it a strong and firm grip, as shown in Figure 2b. SMA (Sub-miniature) is then attached to both ends of the duroid. The attachment was done by fixating screws after drilling holes by the side of the duroid. Care is taken to ensure that the inner conductor of the SMA is placed in contact with the line of the microstrip. The measurement of scattering parameter using the fabricated microstrip was achieved by placing the sample flat on the surface of the microstrip avoiding any air gap between the sample and the microstrip transmission line.

### 2.3. Preparation of OPEFB/PLA Composites

**Figure 3:**
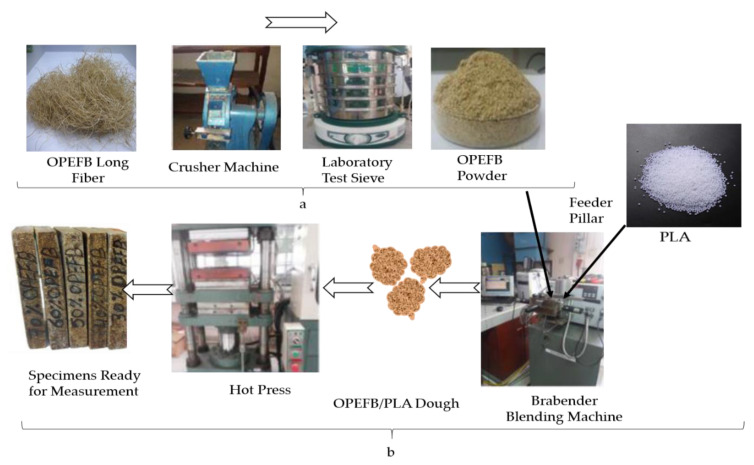
(**a**) Preparation process of OPEFB powder, and (**b**) OPEFB/PLA substrate preparation.

The OPEFB fiber was washed by soaking in distilled water for one day to remove the wax and other impurities. The fiber was rinsed with hot water and acetone before drying at 80 °C for 12 h in an oven [19]. Figure 3a,b shows the procedure for the preparation of the fiber powders and OPEFB/PLA composite substructure preparation, respectively. Figure 3a shows fiber chains ground into powder using a grinding machine (Mainland, Hunan, China) and then sieved using a laboratory test sieve (Endecotts Limited, London, England) to a particle size of 200 μm. Figure 3b displays the preparation of OPEFB/PLA composites via the melt blend technique using the Brabender Internal Mixer machine (GmbH & CO. KG, Duisburg, Germany) with a drive three-phase motor of 18.5 kW, 40 A, 3X400 V, and 50 rpm drive. In this method, the machine is set at the heating temperature of interest and allowed to heat up. The machine is set to heat up to 170 °C (melting point of PLA), the rotation of the rotors was set to 50 min^−1^, polylactic acid (PLA) was added to the blender and mixed for 5 min after which the OPEFB powder was introduced into the blender. The prepared dough was used to make the substrates 3 mm thick by pressing inside a rectangular mold of 10 × 8 cm^2^, which was heated up to 170 °C for 10 min, as illustrated in Table 1.

### 2.4. Measurement of Scattering Parameters

**Figure 4:**
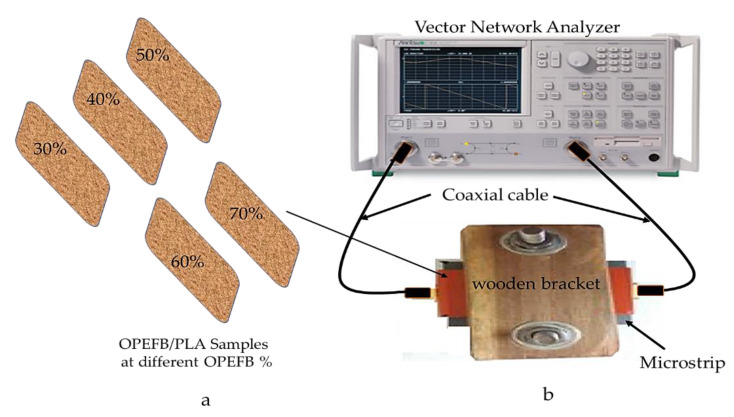
(**a**) Samples ready for measurement, (**b**) microstrip sensor technique measurement set-up.

Measurement of scattering parameters S_11_ and S_21_ for each of the air (without sample) and OPEFB/PLA composites at different percentages of OPEFB was performed by a microstrip transmission line, Figure 4a,b, using the Agilent N5230A PNA-L network analyzer system (Agilent Technologies, Inc., CA, USA) with a commercial measurement (Agilent 85701B, CA, USA) software package [20], at a frequency range from 0.20 to 12 GHz. Measurement of scattering parameters using the fabricated microstrip transmission line was achieved by placing the OPEFB/PLA samples flat, as shown in Figure 4a, on the surface of the microstrip. To avoid the influence of an air gap during the measurement, a wooden bracket was used to apply uniform pressure on the sample, as shown in Figure 4b. The vector network analyzer (VNA) was calibrated by implementing Electronic Calibration modules (N4691-60004) and the accuracy of the VNA depends on the quality of the calibration standards.

### 2.5. Measurement of the Complex Permittivity

**Figure 5:**
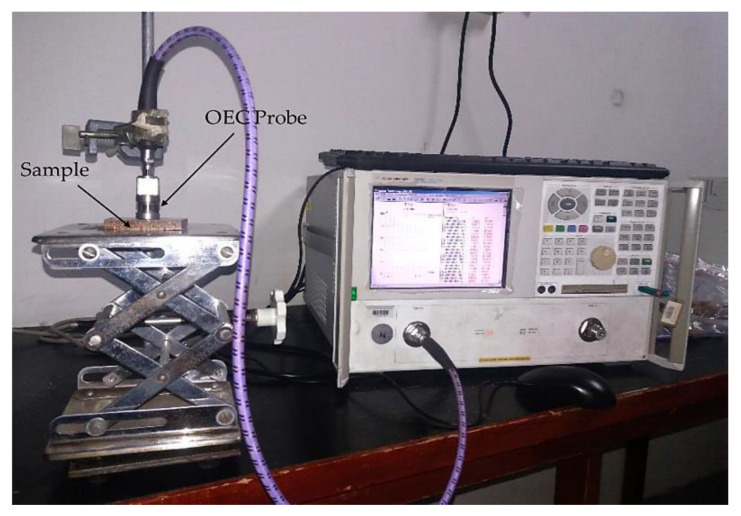
Set-up of relative permittivity measurement using open-ended coaxial probe.

The complex permittivity (dielectric constant and loss factor) of the OPEFB/PLA composites at different percentages of OPEFB filler was measured via the open-ended coaxial probe (OEC) in conjunction with an Agilent N5227A Network Analyzer (Agilent Technologies, Inc. Santa Clara, CA, USA) and commercial measurement software (Agilent 85070B, Agilent Technologies, Inc., Santa Clara, CA, USA), as shown in Figure 5. The complete Agilent measurement kit consists of an Agilent 85070B sensor probe, a mounting bracket, a cable, a 3.5-inch high density shorting block for calibration, adapters, and software for data collection and plotting. The stages of the open-ended coaxial technique calibration used were the open standard air, a short circuit, and distilled water. After complete calibration, Polytetrafluoroethylene (PTFE) was used as the standard material to confirm the calibration before the commencement of measurements of the substrate. The measurement of permittivity is based on the interaction of the external electric field with the electric dipole moment and charges of the materials. When an electric field is applied to a dielectric material, three types of effects occur. The first type is energy reflection, the second type is energy absorbance, and the last type is surface transmittance. These effect types help indicate the material’s electrical properties involved in relative permittivity. Complex permittivity ($\varepsilon_{r}^{*}$) measures the effect of the material on the electric field [19]. The mathematical expression of complex permittivity is given by the following equation:

(1) $$\varepsilon_{r}^{*}=\varepsilon_{r}^{\prime }-j\varepsilon_{r}^{\prime \prime },$$

where $\varepsilon_{r}^{\prime }$ represent the real part of permittivity and $\varepsilon_{r}^{\prime \prime }$ is the imaginary part of permittivity.

### 2.6. Finite Element Method (FEM)

The finite element method (FEM) is one of the most successful frequency-domain computational techniques for electromagnetic simulations. The technique’s main advantage is its ability to treat any kind of geometry and material inhomogeneity without a need to alter the computer code or the computational formulation. That is, it provides geometrical fidelity and unrestricted material treatment [21]. In this research, the FEM technique-based COMSOL software was used to determine the exact transmission S_21_ and reflection S_11_ coefficients of the microstrip line system. The formed microstrip line geometry is designed and connected to a coaxial mode for port boundary conditions. The coaxial mode is the only fundamental mode that is available as a predefined model in COMSOL simulation [22]. Therefore, the electric field vector in coaxial cable in a radial direction and the magnetic field lines are perpendicular so that their lines appear as concentric circles around the center conductor [23].The electric field distributions surrounding the microstrip for OPEFB/PLA composites, where the lines indicate the direction of the field vector from the positive charge to the negative charge. Few steps need to be performed before the simulation process, which is described as follows: creating the geometry, defining physical parameters and boundary conditions, meshing the geometry, solving the model geometry, obtaining the solution, and performing parametric studies.

## 3. Experimental Results and Discussion

### 3.1. Reflection and Transmission Coefficient Measurements

**Figure 6:**
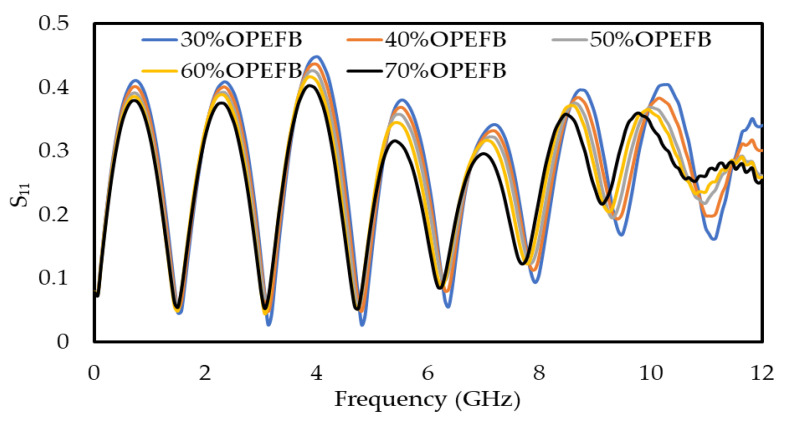
Variation of reflection (S_11_) for OPEFB/PLA composite at different percentages of OPEFB.

**Figure 7:**
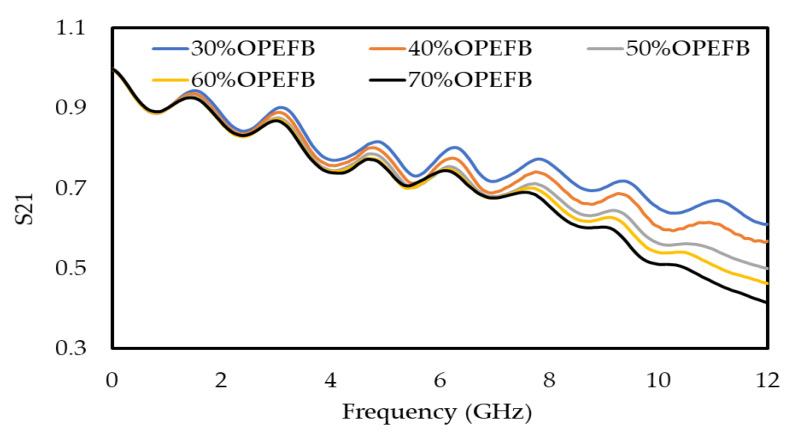
Variation in transmission (S_21_) for OPEFB/PLA composites at different percentages of OPEFB filler.

Composites with good properties can be obtained when the filler is well dispersed in the matrix. Moreover, the filler and the matrix should be compatible [24], thus the filler (OPEFB) helps to control the general properties of the OPEFB/PLA composites. The effects of OPEFB loading on the S_11_ and S_21_ on the frequency were studied and clarified, as shown in Figure 6 and Figure 7, respectively, by using a microstrip line technique. The decrease in S_11_ magnitude as the percentage of OPEFB increases is shown in Figure 6, and the difference in magnitude of S_11_ is distinguishable from one sample to another. The magnitude of S_11_ at 1 GHz of OPEFB/PLA composites with different % of OPEFB fillers are 0.0836, 0.0821, 0.081, 0.079, and 0.078 for 30%, 40%, 50%, 60%, and 70% OPEFB, respectively. The magnitude of S_11_ at 12 GHz are 0.335, 0.301, 0.273, 0.262, and 0.251 for 30%, 40%, 50%, 60%, and 70% OPEFB, respectively. Figure 7 shows the effect of frequency and OPEFB loading on the S_21_ magnitude. It is observed that the S_21_ magnitude decreases with an increase in the frequency and decreased with an increase in the percentage of OPEFB filler. Furthermore, if the materials have a higher loss factor, they tend to absorb more energy of the electromagnetic waves that were propagating through the sample resulting in a reduced S_21_ magnitude. The magnitude of S_21_ at 1 GHz of OPEFB/PLA composites with different % of OPEFB fillers are 0.985, 0.984, 0.975, 0.974, and 0.973 for 30%, 40%, 50%, 60%, and 70% OPEFB, respectively. The magnitude of S_21_ at 12 GHz are 0.613, 0.585, 0.501, 0.475, and 0.424 for 30%, 40%, 50%, 60%, and 70% OPEFB, respectively.

### 3.2. Complex Permittivity of OPEFB/PLA Composites

**Figure 8:**
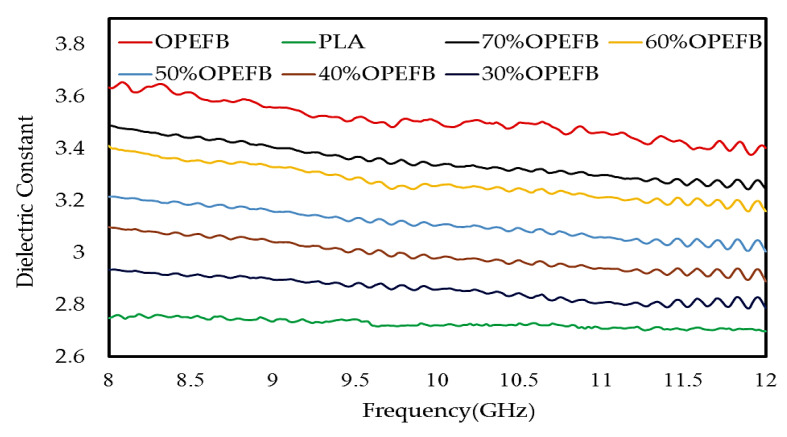
Variation in dielectric constant of OPEFB/PLA composites at different percentages of OPEFB filler.

**Figure 9:**
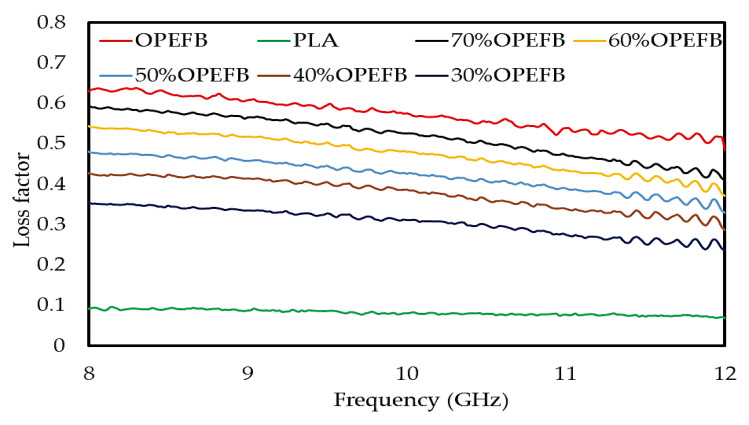
Variation in loss factor of OPEFB/PLA composites at different percentages of OPEFB filler.

The complex permittivity (dielectric constant ($\varepsilon_{r}^{\prime }$) and loss factor ($\varepsilon_{r}^{\prime \prime })$) of the OPEFB/PLA composites at different percentages of OPEFB filler was measured using the OEC technique. The fiber size of the OPEFB was chosen to be 200 μm to obtain the highest loss factor in order to increase the composite absorption property. The variation for the different OPEFB/PLA composites at the 8–12 GHz range is presented in Figure 8 and Figure 9 for the dielectric constant ($\varepsilon_{r}^{\prime }$) and loss factor ($\varepsilon_{r}^{\prime \prime }$), respectively. The $\varepsilon_{r}^{\prime }$ and $\varepsilon_{r}^{\prime \prime }$ depend on the contributions of the orientation, interface, as well as electronic and atomic polarization in the materials [14]. The interfacial polarization arose due to the differences in polarization or conductivities of the matrix and fibers. Both the interfacial and the orientation polarization of a composite depend on the concentration of fibers. In mixture composites, the significant increase in $\varepsilon_{r}^{\prime }$ and $\varepsilon_{r}^{\prime \prime }$ with an increase in OPEFB fiber loading was attributed to the increase in interfacial and orientation polarization resulting from the occurrence of polar categories of cellulose in natural fibers.

Higher interface and orientation polarization led to the increment in $\varepsilon_{r}^{\prime }$ and $\varepsilon_{r}^{\prime \prime }$. This may be a result of the hydrophilic nature of cellulose fibers which absorbs moisture content from the air leading to increased conductivity of the polymer materials [24]. Figure 8 and Figure 9 showed a variation in $\varepsilon_{r}^{\prime }$ and $\varepsilon_{r}^{\prime \prime }$ for all OPEFB/PLA composites. It is observed that the value of the dielectric constant and loss factor is higher for the composites with a higher content of OPEFB filler. Figure 8 shows a variation in dielectric constant from 2.746 to 3.486 between PLA and the OPEFB fiber. A careful observation in Figure 8 showed that 70 wt% of OPEFB filler has successfully enhanced the dielectric constant of the composites. The percentage increase of OPEFB would strengthen the polarization of OPEFB/PLA composites which in turn would increase the $\varepsilon_{r}^{\prime }$ and $\varepsilon_{r}^{\prime \prime }$. While Figure 9 shows the effect of the different percentages of OPEFB on the imaginary part $\varepsilon_{r}^{\prime \prime }$ of OPEFB/PLA composites, the microwave frequency showed a variation in loss factor from 0.090 dB to 0.5941 dB at the highest percentage of 70 wt% OPEFB filler. The dielectric properties obtained from the open-ended coaxial prob were required as inputs to FEM to calculate the S_11_ and S_21_ of the samples.

### 3.3. Electric Field Distribution Surrounding the Microstrip Sensor

**Figure 10:**
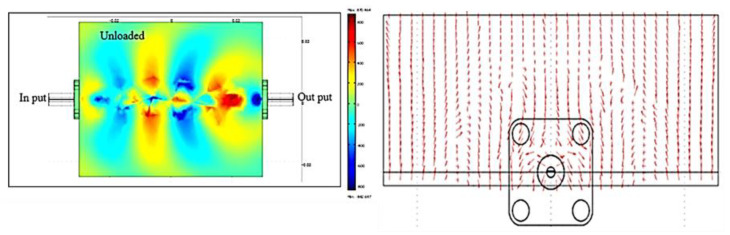
Electric field distribution and intensity plot for the unloaded microstrip.

**Figure 11:**
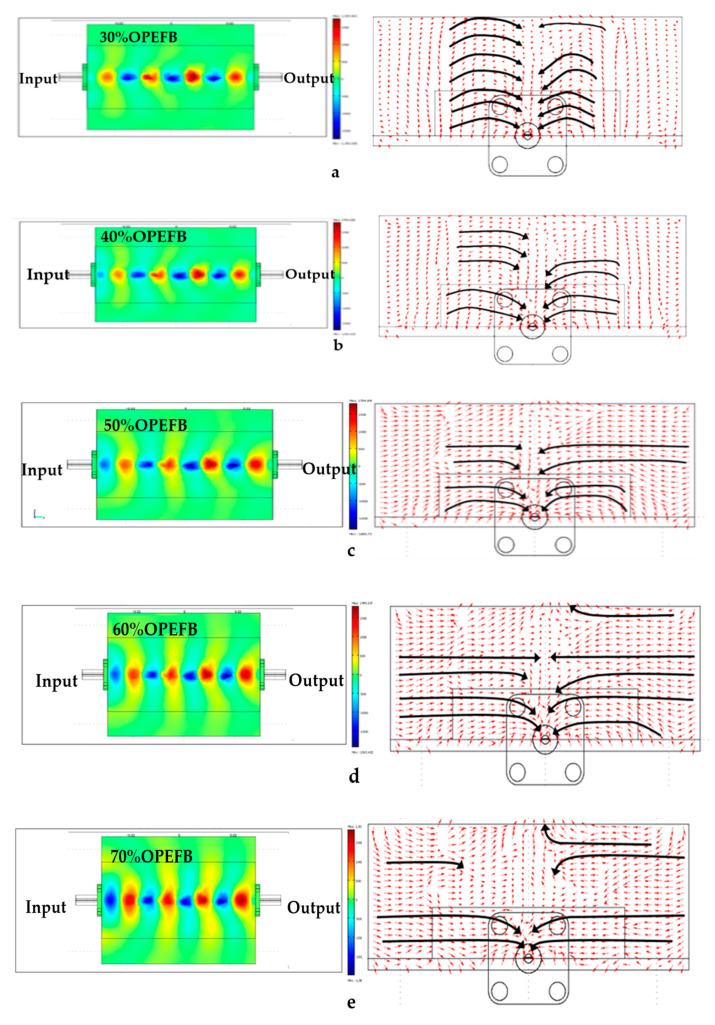
Electric field distribution and intensity plot for the different OPEFB/PLA composites (**a**) 30 wt%, (**b**) 40 wt%, (**c**) 50 wt%, (**d**) 60 wt%, and (**e**) 70 wt% of OPEFB.

This section presents the electric field distribution surrounding the microstrip sensor for OPEFB/PLA composites shown in Figure 10 and Figure 11 obtained from FEM simulations. The arrows indicate the direction of the field vector from the positive charge to the negative charge. Collectively, the arrows form the overall shape of the field vector surrounding the sensor which is represented with the bold line. It should be noted, however, that the bold line does not represent the strength of the electric field but only serves to show the overall shape of the electric field. The electric field is a function of many parameters, including frequency, permittivity, and permeability of the surrounding material [25,26]. Figure 10 illustrates the electric field distribution at the unloaded microstrip (without sample), showing that most of the uniform field distribution of the electric fields converged at the top of the conducting strip.

To calculate the magnitude of transmission S_21_ and reflection S_11_ coefficient parameters of OPEFB/PLA composites using (FEM) simulation by the COMSOL software, the solution time is strongly influenced by mesh properties such as element quality geometry, conformity, and mesh density. Therefore, an appropriate approximation of the problem domain is required for the geometry conformity of the area defined by the mesh elements. In this research, the FEM simulation results of the electric field distribution of the microstrip sensor covered with OPEFB/PLA composites are illustrated in Figure 11a–e in which the arrow and the colorful shape represent the direction, intensity, and shape of the electric field distribution. The sample results show the decrement in the intensity of wave propagation as the OPEFB filler content increases, which is dependent on electric permittivity values that change as the filler ratio changes, thus agreeing with experimental S_21_ and S_11_ measurement results. As expected, higher loss material has higher absorption and thus lower transmission of the electromagnetic waves through the OPEFB/PLA composites material. Thus, the higher the dielectric constant and loss factor, the less uniform the distribution of the electric field is observed due to scattering as shown in Figure 11d,e [27,28]. On the other hand, the higher the content of OPFEB filler, the higher the absorption loss, and thus the lower the intensity of the propagating wave as shown in the simulation, which is in complete agreement with the experimental loss factor results obtained for the different percentages of OPEFB filler. The simulation results showed that the electric field radiation pattern distributed over the OPEFB/PLA composites is dependent on complex permittivity values which determines the direction of the electric field [29]. Minimization of the discretization error and achieving accurate solutions can be assured by having the mesh with density and size that are sufficiently high and small, respectively [22]. Shown in Table 2 are the relative errors of measurement and simulation of both of S_11_ and S_21_.

### 3.4. Calculated Attenuation

The attenuation is caused by absorption and scattering of wave power. Therefore, the attenuation or transmission loss is defined as the ratio of input and output powers [30]. The attenuation values of the OPEFB/PLA composites can be calculated using the results of transmission coefficients S_21_, which is obtained via the vector network analyzer by applying the following Equation [10]:

(2) $$\mathrm{Attenuation}\;\left(\mathrm{dB}\right)=-20\;\log\;\left(S_{21}\right).$$

**Figure 12:**
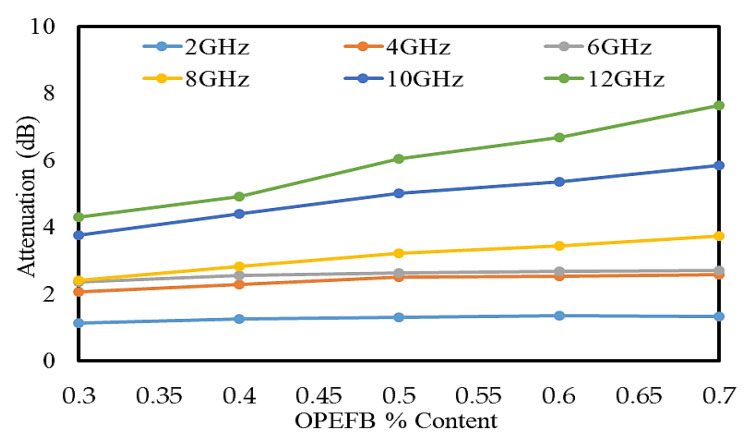
Calculated attenuation of OPEFB/PLA composites at different OPEFB wt% filler.

The calculated results showed that the attenuation of the OPEFB/PLA composites increases as the filler content increases. This confirms the proportional relationship between the attenuation outcome of the OPEFB/PLA composites and the filler composition. Figure 12 illustrates that the lowest attenuation value was calculated for the 30 wt% OPEFB particle filler, whereas the highest attenuation was calculated for the 70 wt% OPEFB particle filler. It is inferred from the results that the attenuation of OPEFB/PLA composites with different wt% of OPEFB increased with increase of the OPEFB filler and frequency. An addition of OPEFB composition was found to enhance the microwave properties of the OPEFB/PLA composites from a low loss material to a high loss material suitable for microwave attenuation. For the 30 wt% OPEFB particle filler, the attenuation at 2 GHz is 1 dB, the attenuation continued to increase to 4.456 dB as the frequency increases to 12 GHz. The attenuation trend did not change with the (40, 50, 60, and 70) wt% OPEFB particles filler at (2, 4, 6, 8, 10, and 12) GHz, respectively. The 40 wt% OPEFB/PLA composites have an attenuation of 1.182 dB at 2 GHz, which increased to 2.201 dB at 4 GHz, 2.352 dB at 6 GHz, 2.813 dB at 8 GHz, 4.213 dB at 10 GHz, and 4.987 dB at 12 GHz. The attenuation for the 70 wt% OPEFB filler at 2 GHz is 1.250 dB, at 4 GHz is 2.511 dB, at 6 GHz is 2.782 dB, at 8 GHz is 3.821 dB, at 10 GHz is 5.831 dB, and at 12 GHz the attenuation is 7.735 dB.

### 3.5. Microwave Absorption

When an electromagnetic wave (EMW) passes through a lossy medium, such as a shield, its amplitude decreases due to the thickness and material absorption [31]. Furthermore, the EM wave absorption of the polymer-based composites depends on many factors, such as the dielectric constant and scattering parameter [24]. Moreover, the ideal performance of one EM wave absorbing material is mainly attributed to two factors: impedance matching and EM wave attenuation. Therefore, the power loss magnitude (P loss) for the microstrip is defined as follows:

(3) $$P_{loss}\left(unloaded\right)=1-{\left(S_{11}\right)}^{2}-{\left(S_{21}\right)}^{2}.$$

Nevertheless, the material absorption loss of OPEFB/PLA composites can be calculated from the power loss difference of a hollow and sample-loaded microstrip.

(4) $$P_{loss}\left(sample\right)=1-{\left(S_{11}\right)}^{2}sample-{\left(S_{21}\right)}^{2}sample,$$

(5) $$Absorption=P_{loss}\left(sample\right)-P_{loss}\left(unloaded\right).$$

**Figure 13:**
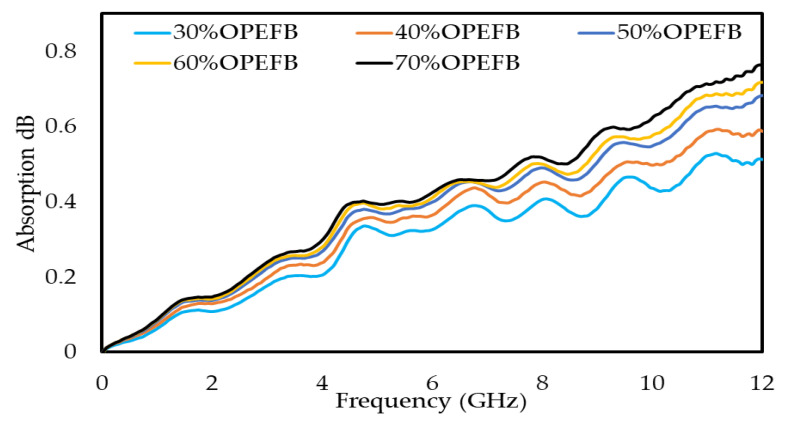
Calculated absorption loss of different compositions of OPEFB/PLA composites at various percentages of OPEFB filler.

For OPEFB/PLA composites, the values of absorption loss at 8 GHz are (0.407, 0.452, 0.489, 0.499, and 0.515) dB for (30, 40, 50, 60, and 70) wt% OPEFB, respectively. The magnitudes of absorption loss at 12 GHz are (0.512, 0.587, 0.683, 0.718, and 0.763) dB for (30, 40, 50, 60, and 70) wt% OPEFB, respectively. The results show that the absorption loss increased with increasing frequency and percentage of the filler, as shown in Figure 13.

### 3.6. Comparison of Measured and Simulated Scattering Parameters

**Figure 14:**
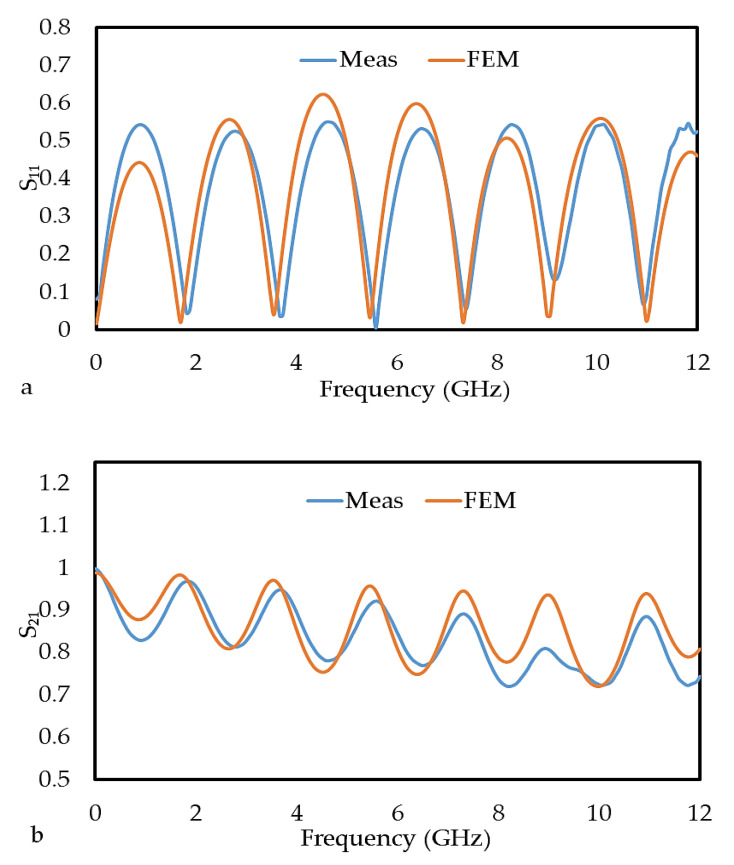
The magnitudes of (**a**) S_11_ and (**b**) S_21_ for both the measurement and FEM simulation method of the unloaded microstrip line.

The S-parameters (S_11_ and S_21_) measurement results of an unloaded microstrip line and FEM simulation in the frequency range from 0 GHz to 12 GHz are shown in Figure 14a,b. As expected for the unloaded microstrip, the value of the transmission coefficient is higher than the reflection coefficient. The accuracy of the S_11_ and S_21_ values can be determined by calculating the relative error for the measurement data [32] as follows:

(6) $$\mathrm{The}\;\mathrm{relative}\;\mathrm{error}\;\mathrm{of}\;S_{11},S_{21}=\frac{S_{11},S_{21}\left(\mathrm{measurement}\right)-S_{11},S_{21}\left(\mathrm{FEM}\right)\;}{S_{11},S_{21}\left(\mathrm{measurement}\right)}.$$

Note that the mean relative errors of S_21_ are calculated by replacing S_11_ with S_21_ in Equation (6). The mean relative errors between the measured and FEM values for S_11_ and S_21_ were 0.478 and 0.049, as listed in Table 3. The high error in S_11_ was due to the effect of multiple reflections not considered in the FEM simulation, which usually requires a large number of small meshes at the interface between the microstrip and the coaxial cable.

**Figure 15:**
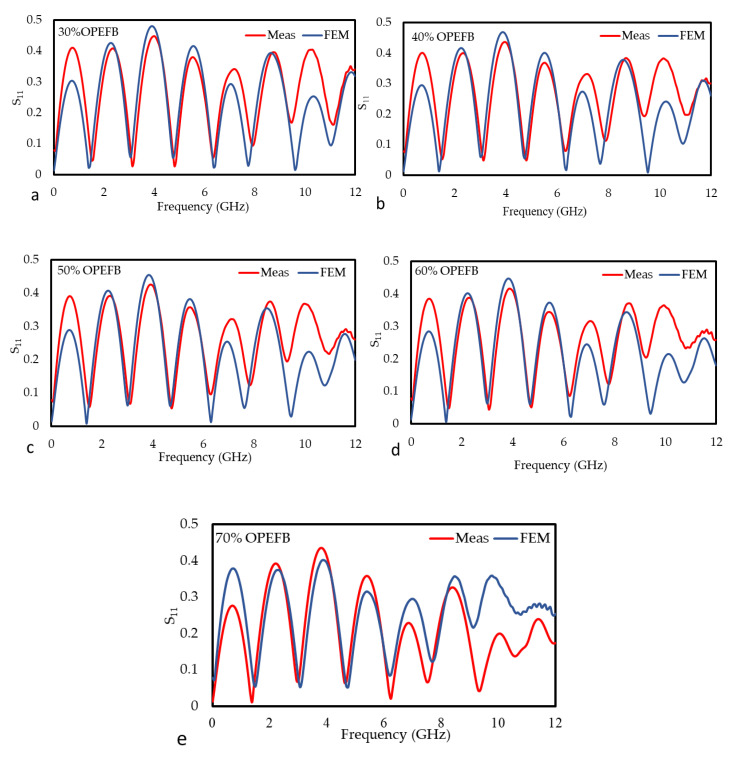
Variation in the magnitude of S_11_ for the different OPEFB/PLA composites (**a**) 30 wt%, (**b**) 40 wt%, (**c**) 50 wt%, (**d**) 60 wt%, and (**e**) 70 wt% OPEFB for both the measurement and FEM simulation.

**Figure 16:**
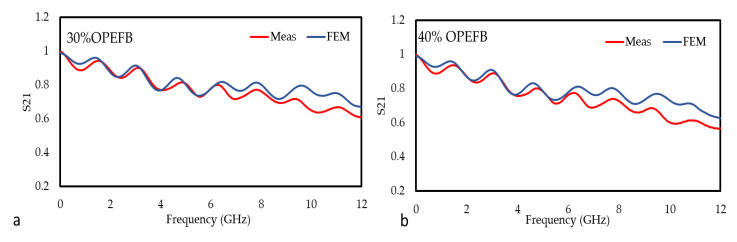

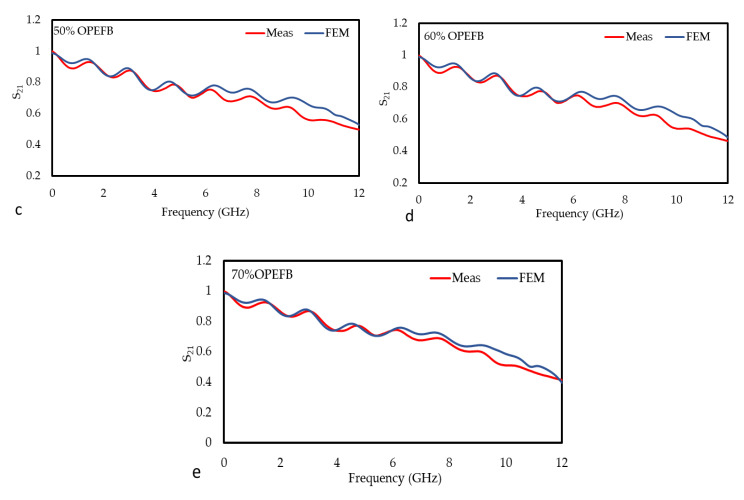
Variation in the magnitude of S_21_ for the different OPEFB/PLA composites (**a**) 30 wt%, (**b**) 40 wt%, (**c**) 50 wt%, (**d**) 60 wt%, and (**e**) 70 wt% OPEFB for both the measurement and FEM simulation.

The graphs in Figure 15 and Figure 16 are the comparison between the measured and simulated (FEM) values of the S_11_ and S_21_ for the OPEFB/PLA composites, respectively. In general, there is a good agreement between the measured and simulated for all the composites. Table 2 shows the mean relative error of S_11_ and S_21_ and it can be observed that the lowest mean relative error of the S_11_ value for OPEFB/PLA composites is at 50 wt% OPEFB, while the highest value is at 30 wt% OPEFB. The lowest mean relative error of S_21_ value is at 70 wt% OPEFB, while the highest value is at 40 wt% OPEFB. The magnitude of S_21_ decreased with increasing OPEFB filler content, where the 70 wt% OPEFB percentage filler sample was found to have the lowest S_21_. These results are in agreement with the impedance mismatch theory where materials with the highest permittivity show lower transmission coefficient values [33]. Therefore, the magnitudes of S_21_ at 12 GHz are (0.610, 568, 0.499, 0.463, and 0.415) dB for (30, 40, 50, 60, and 70) wt% OPEFB, respectively.

## 4. Error Analysis

Several factors that affect measurement accuracy have been determined using the proposed methods, such as the uncertainty in measured S-parameters. Several other components specified for random errors in measurement are the connectors between the microstrip line and network analyzer, which can make the system sensitive to movement. Another random error that appeared in the air gap between the internal surface of the sample and the external surface of the microstrip line was minimized by the pressing on the sample from the top. Furthermore, keeping the whole system clean and stable minimizes the errors, whereas systematic errors from the imperfections of the system were reduced by careful calibration of the whole measurement system before the practical measurements.

## 5. Conclusions

In this research, the OPEFB/PLA composites with 30–70 wt% of OPEFB filler were successfully prepared based on the blending technique using a Brabender Internal Mixer machine. The effectiveness of the composites at various ratios of OPEFB filler at a frequency range of 0.20–12 GHz on both of the absorption and attenuation was studied. The attenuation and absorption of OPEFB/PLA composites were calculated using the results obtained from the measurement of reflection S_11_ and transmission S_21_ coefficients of the composites using the microstrip transmission line technique. Results obtained for the measurement of reflection coefficients revealed that S_11_ increases with increase in OPEFB particle filler for all samples used in this study and vice versa for the transmission S_21_ coefficients. The calculated attenuation for the 30 wt% OPEFB filler showed that attenuation was lowest at 2 GHz and the highest attenuation was recorded at 12 GHz. The absorption of the different OPEFB/PLA composites showed that the magnitude of absorption continues to increase as the filler content increases until it reached the highest value. It is observed from the results that the value of the dielectric constant and loss factor is higher for the OPEFB/PLA composites with a higher content of OPEFB filler. The dielectric constant increased from 2.746 dB to 3.486 dB, while the loss factor increased from 0.090 dB to 0.5941 dB at the highest percentage of 70 wt% OPEFB filler. The dielectric properties obtained from the open-ended coaxial probe were required as inputs to FEM to calculate the S_11_ and S_21_ of the samples. The comparison between the S_11_ and S_21_ for the measured and simulated (FEM) values for the microstrip transmission line technique (unloaded) was studied and the mean relative error between the measured and FEM for S_11_ and S_21_ was found to be 0.478 and 0.049, respectively. The results of S_11_ and S_21_ calculated by FEM simulation was found to agree with the magnitudes of the reflection and transmission coefficients, S_11_ and S_21_, measured by the microstrip transmission line technique with little relative error.

## Figures and Tables

**Table 1:** OPEFB/PLA composites at different percentages of fiber.

OPEFB		PLA		Total Mass (g)
Weight (%)	Mass (g)	Weight (%)	Mass (g)	
30	13.50	70	31.50	45
40	18.00	60	27.00	
50	22.50	50	22.50	
60	27.00	40	18.00	
70	31.50	30	13.50	

**Table 2:** The mean relative error of S_11_ and S_21_ for OPEFB/PLA composite.

OPEFB	Relative Error	
	S_11_	S_21_
30%	0.301	0.051
40%	0.280	0.068
50%	0.273	0.054
60%	0.273	0.053
70%	0.298	0.046

**Table 3:** The mean relative error of S_11_ and S_21_ for unloaded microstrip line.

Sample	Relative Error	
	S_11_	S_21_
AIR	0.478	0.049
